# Limiting Uncertainty Relations in Laser-Based Measurements of Position and Velocity Due to Quantum Shot Noise

**DOI:** 10.3390/e21030264

**Published:** 2019-03-08

**Authors:** Andreas Fischer

**Affiliations:** Bremen Institute for Metrology, Automation and Quality Science (BIMAQ), University of Bremen, Linzer Str. 13, 28359 Bremen, Germany; andreas.fischer@bimaq.de; Tel.: +49-421-64600

**Keywords:** optical metrology, shot noise limit, measurement uncertainty, Fisher information, estimation theory, Cramér-Rao inequality

## Abstract

With the ongoing progress of optoelectronic components, laser-based measurement systems allow measurements of position as well as displacement, strain and velocity with unbeatable speed and low measurement uncertainty. The performance limit is often studied for a single measurement setup, but a fundamental comparison of different measurement principles with respect to the ultimate limit due to quantum shot noise is rare. For this purpose, the Cramér-Rao bound is described as a universal information theoretic tool to calculate the minimal achievable measurement uncertainty for different measurement techniques, and a review of the respective lower bounds for laser-based measurements of position, displacement, strain and velocity at particles and surfaces is presented. As a result, the calculated Cramér-Rao bounds of different measurement principles have similar forms for each measurand including an indirect proportionality with respect to the number of photons and, in case of the position measurement for instance, the wave number squared. Furthermore, an uncertainty principle between the position uncertainty and the wave vector uncertainty was identified, i.e., the measurement uncertainty is minimized by maximizing the wave vector uncertainty. Additionally, physically complementary measurement approaches such as interferometry and time-of-flight positions measurements as well as time-of-flight and Doppler particle velocity measurements are shown to attain the same fundamental limit. Since most of the laser-based measurements perform similar with respect to the quantum shot noise, the realized measurement systems behave differently only due to the available optoelectronic components for the concrete measurement task.

## 1. Introduction

### 1.1. Motivation

Optical measurement systems use light to carry and transport information with the fastest possible speed—with light speed! Furthermore, to measure (or to quantify) means to compare with a unit, and for optical measurements the measurand is compared with units originating from photon characteristics, which enables fast and precise measurements (to a certain extent).

Considering dimensional measurements with visible light, the reference is the photon wavelength from 380 nm to 750 nm that enables measurement resolutions in the micro- or nanometer range and even below. Optical position or distance measurements have driven science forward, e.g., using the frequency comb technique [1] (Nobel prize in physics 2005) allowing 100 million measurements per second with sub-millimeter resolution [2], using the stimulated emission depletion technique [3] (Nobel prize in chemistry 2014) allowing microscopy with visible light below Abbe’s diffraction limit in the nanometer range [4] as well as using interferometry and squeezed light techniques [5] that enabled the detection of gravitational waves [6] (Nobel prize in physics 2017) by measuring distance variations in the order of attometer in milliseconds. The different principles for position and distance measurements are reviewed in [7], which are implemented in state-of-the-art measurement systems that are commercially available.

The first derivative of the position with respect to the time is the velocity, which is of outstanding importance for instance in the understanding and characterization of fluid mechanics including the riddle of turbulence [8]. Current reviews for micro fluidic flow measurements [9] and meso- and macroscopic optical flow velocity field measurements based on Mie scattering [10] illustrate the huge application potential, e.g., for aerodynamic studies in turbomachinery [11,12], for thermoacoustic phenomena in flame flows [13,14], for transient two-phase flow phenomena in fast fuel injections shorter than one millisecond [15,16]. Further flows of interest are large scale flows such as occurring on the sun surface with velocity fluctuations up to ±1000 m/s [17] or small scale flows in micro channels in the order of mm/s [18]. Regarding the velocity measurement of a solid object, velocities of far planets are demonstrated for instance to be measurable with resolutions down to a centimeter per second by using the frequency comb technique [19].

The occurring question for laser-based measurements in particular regarding position (or distance) and velocity information is: Does a natural, fundamental limit of measurability exist and how to describe it?

### 1.2. State of the Art

Light is an electromagnetic phenomenon that follows the rules of quantum mechanics. As such, the light particles (photons) satisfy Heisenberg’s uncertainty principle, which states that the standard uncertainty $\sigma_{x}$ of the position *x* and the standard uncertainty $\sigma_{p}$ of the momentum *p* of a photon fulfill the inequality [20,21]

(1) $$\begin{array}{c} \sigma_{x}\cdot \sigma_{p}\geq \frac{h}{4\pi } \end{array}$$

with *h* as the Planck constant. Similar uncertainty principles can be derived for energy and time, frequency and time, as well as amplitude (or photon number) and phase [22]. Please note that the minimal achievable standard uncertainty for average values of, for instance, position or momentum of *N* uncorrelated photons is a factor of $1/\sqrt{N}$ smaller than given by Equation (1).

A special case that minimizes Heisenberg’s inequality relation is coherent light with a constant intensity, where the number of photons obeys a Poissonian distribution [23]. For this reason, the resulting photon shot noise is also termed a quantum limit in optics. Hence, Heisenberg’s uncertainty principle as well as a Poissonian photon statistic can be used as a starting point to derive fundamental limits of laser-based measurements.

While the application of Heisenberg’s uncertainty inequality requires a detailed physical understanding, the respective measurement limit can be determined in a more formalized manner with an information theoretic approach on the basis of a light intensity signal with a Poissonian photon number distribution. The required tool from information theory is the Cramér-Rao bound (CRB), which gives the lowest possible variance of any unbiased estimator, i.e., the lowest achievable measurement uncertainty squared for the case of no systematic error [24]. As a result, the CRB of a measurand θ leads straightforwardly to the desired measurement limit of the measurand:

(2) $$\begin{array}{c} \mathrm{Var}\left(\hat{\theta }\right)\geq \mathrm{CRB}\left(\theta \right). \end{array}$$

Please note that a single unknown quantity is considered here as measurand, and $\hat{\theta }$ is an unbiased estimator for the measurand θ.

For a single unknown, the CRB in the case of unbiased estimation is the inverse of the Fisher information I(θ), so that Equation (2) becomes

(3) $$\begin{array}{c} \mathrm{Var}\left(\hat{\theta }\right)\cdot I\left(\theta \right)\geq 1. \end{array}$$

Since the Fisher information is the curvature of the relative information entropy (Kullback-Leibler divergence), the found inequality states an uncertainty principle on the basis of entropy (measure of the average information content in the signal): The higher/lower the Fisher information, the lower/higher is the achievable measurement uncertainty squared. However, while the application of the CRB for identifying fundamental measurement limits is well-known for signals with Gaussian noise [25,26], the CRB with respect to signals with Poissonian noise is hidden in mathematical textbooks [27] and is rarely applied.

### 1.3. Aim and Structure of the Article

The aim of the article is first to introduce in the identification of fundamental measurement limits due to the quantum shot noise of laser-based measurements by calculating the CRB. Furthermore, historical and recent achievements are reviewed with respect to laser-based position, displacement, strain and velocity measurements.

In Section 2, the fundamentals for applying the Cramér-Rao inequality are described. The respective measurement limits in laser-based position as well as displacement, strain and velocity measurements are reviewed in Section 3 and Section 4, respectively. Finally, the main conclusions and an outlook are given in Section 5.

## 2. Application of the Cramér-Rao Inequality

The CRB [28,29] follows when the well-known Cauchy-Schwarz inequality is applied to the product of the variance of the estimator $\hat{\theta }=f\left(\vec{x}\right)$ of an unknown quantity θ in a signal $\vec{x}\in R^{l}$ with l∈N elements, and the variance of the respective score function $\frac{\partial \ln p(\vec{x},\theta )}{\partial \theta }$, which is derived from the likelihood function *p*, i.e., the joint probability function for the realization of a specific signal as a function of the unknown θ. As a result, a lower bound for the minimal achievable variance of all unbiased estimators is obtained from the Fisher information I(θ):

(4) $$\begin{array}{c} \mathrm{Var}\left(\hat{\theta }\right)\geq {\left(I\left(\theta \right)\right)}^{-1} \end{array}$$

with

(5) $$\begin{array}{c} I\left(\theta \right)=E\left({\left(\frac{\partial \ln p(\vec{x},\theta )}{\partial \theta }\right)}^{2}\right). \end{array}$$

The derivation of the CRB contains the assumption

(6) $$\begin{array}{c} E\left(\frac{\partial \ln p(\vec{x},\theta )}{\partial \theta }\right)=0, \end{array}$$

which is known as regularity condition [26,27]. This regularity condition is fulfilled for white Gaussian and Poissonian noise with constant variance. By applying the regularity condition, the calculation of the Fisher information is also possible with the second derivative of the log-likelihood function instead of squaring the first derivative [26]:

(7) $$\begin{array}{c} E\left({\left(\frac{\partial \ln p(\vec{x},\theta )}{\partial \theta }\right)}^{2}\right)=E\left(-\frac{\partial^{2}\ln p\left(\vec{x},\theta \right)}{\partial \theta^{2}}\right). \end{array}$$

Please note that only the case of a single unknown quantity is considered here, which is a best case scenario and usually sufficient for the estimation of the measurement limit. If multiple unknown quantities exists, the quantity θ as well as the respective CRB become a vector and the inverse of the Fisher-information matrix needs to be evaluated [26,27]. In that case, not the scalar but the matrix version of the Cauchy–Schwarz inequality is required [30]. However, the variance of the unknown quantity remains bounded by the same limit since the available information decreases. In order to estimate the ultimate lower bound and for the sake of simplicity, multiple unknown quantities are not considered here.

The CRB allows determining the measurement uncertainty limit, to characterize the efficiency of the signal processing algorithm by evaluating the ratio of the CRB to the estimator variance (1 best, 0 worst) and to identify fundamental uncertainty principles.

### 2.1. Entropic Uncertainty Principles

The lower bound of the estimator variance in Equation (4) can be considered as a fundamental entropic uncertainty relation between the minimal achievable measurement uncertainty squared and the information with respect to the measurand that is contained in a signal. Note that the uncertainty squared is a noise signal power. When both sides of Equation (4) are multiplied with the measurement time *T*, an uncertainty relation between the noise signal energy and the average information rate becomes visible:

(8) $$\begin{array}{c} \mathrm{Var}\left(\hat{\theta }\right)\cdot T\geq {\left(\frac{I(\theta )}{T}\right)}^{-1}. \end{array}$$

This relation can also be interpreted as an uncertainty principle between the measurement uncertainty squared and the measurement time [31]. Considering signals not over time but over space such as images, the similar holds with respect to the spatial dimensions. Hence, the product between the minimal achievable measurement uncertainty squared and the resolution of the measurement over the time or space axis is ultimately limited by the average information rate or density, respectively. However, there is no standard in measurements regarding the documentation of noise signal energy, but only regarding the square root of the noise signal power, which is the measurement uncertainty. For this reason, the article is focused on the uncertainty principle formulated with respect to the measurement uncertainty in Equation (4).

### 2.2. Guide to the Expression of Uncertainty in Measurement

The existing guide to the expression of uncertainty in measurement (GUM) [32] is internationally accepted and perfectly compatible with an analysis of the CRB. According to the GUM, known systematic errors have to be corrected and are not considered further. Unknown systematic errors are modeled as random errors, so that only random errors remain. As an example, the behavior of the Fisher information and the resulting CRB for typical unknown systematic errors such as an offset or a linear drift as random error was recently studied [33]. Finally, the standard deviation of the measurand that results from the random error contributions is determined as standard measurement uncertainty, and the minimal achievable measurement uncertainty squared is the CRB with respect to the considered signal model (including noise).

A CRB can be determined for biased estimation (measurement with systematic error) and unbiased estimation (measurement with no systematic error) [24,27]. While the CRB for biased estimators depends on the estimator’s bias, all unbiased estimators fulfill the same CRB. Since a bias is a systematic error that can be eliminated with a calibration, only the unbiased case is of interest here, leading to the lowest possible measurement uncertainty in accordance with the GUM. For this reason, the CRB that is valid for all unbiased estimators is given in Equation (4).

Note that optimized biased estimators were studied for attaining a lower mean square error than with unbiased estimation [34,35,36,37,38,39,40]. However, the measurement uncertainty is *not* reduced, because a bias can be determined with a calibration and therefore should not be included in the discussion of the measurement uncertainty according to the GUM.

### 2.3. Beyond the Classical CRB

The original CRB was found independently by Fréchet [41], Darmois [42], Rao [28] and Cramér [29,43] between 1943 and 1946. The proof of the attainability followed by Wijman in 1973 [44]. A spectral formulation of the CRB for Gaussian noise was derived by Zeira and Nehorai in 1990 [45], which was complemented by Fischer and Czarske in 2015 [31].

Many extensions of the CRB took place. In 1946 Bhattacharrya proposed to consider also higher order derivatives of the log-likelihood function [46]. Wolfowitz studied the CRB enhancement from a single estimation to a sequence of estimations in 1947 [47,48]. Instead of the Cauchy-Scharz inequality, Barankin applied the more general Hölder inequality in 1949 to derive a lower bound that is not only based on the second central moment, but an arbitrary absolute central moment [49,50]. In 1951, Chapman and Robins derived a lower bound without the regularity conditions of the Fisher information, which are required to derive the CRB [51,52]. One year later, Fraser and Guttman accomplished the same for the Bhattacharrya bound [53]. Kullback and Leibler found a lower bound on the basis of the Kullback-Leibler divergence instead of the Fisher information in 1951, also without the need for certain regularity conditions [54,55]. Overviews of the different lower bounds are presented for instance in [56,57,58]. Furthermore, Gart enhanced the CRB analysis from deterministic to stochastic unknown parameters in 1959 [59], and Simonov derived the CRB for functional parameters in 2014 [60].

Due to the close relation between the CRB and the minimal achievable measurement uncertainty of a measurand that is obtained from a signal with superposed noise, and since the original CRB is the lowest and easiest lower bound to derive, the present article focuses on a derivation of measurement limits using the original scalar CRB for the case of unbiased estimation from Equation (4).

### 2.4. CRB for Signals in White Noise

In order to derive the CRB for the unknown measurand θ that is estimated from the signal $\vec{x}={\left(N\left[1\right],\ldots ,N\left[m\right],\ldots ,N\left[l\right]\right)}^{T}$, the likelihood function *p* needs to be known. Please note that the signal is written in the unit number of photons and the aim is to derive the CRB due to quantum shot noise so that white Poissonian noise (WPN) can be assumed. Hence, the likelihood function is

(9) $$\begin{array}{c} p\left(\vec{x},\theta \right)=\prod_{m=1}^{l}\frac{\bar{N}{\left[m\right]}^{N[m]}}{N[m]!}e^{-\bar{N}\left[m\right]}. \end{array}$$

The symbol $\bar{N}\left[m\right]$ denotes the noise-free mean of a signal sample. The log-likelihood function then reads

(10) $$\begin{array}{c} \ln p\left(\vec{x},\theta \right)=\sum_{m=1}^{l}N\left[m\right]\ln\left(\bar{N}\left[m\right]\right)-\ln\left(N\left[m\right]!\right)-\bar{N}\left[m\right] \end{array}$$

and the first derivative is

(11) $$\begin{array}{cc} E\left(\frac{\partial \ln p(\vec{x},\theta )}{\partial \theta }\right) & =E\left(\sum_{i=1}^{l}\frac{\partial \ln p(\vec{x},\theta )}{\partial \bar{N}\left[i\right]}\cdot \frac{\partial \bar{N}\left[i\right]}{\partial \theta }\right)\\ & =E\left(\sum_{i=1}^{l}\left(\frac{N[i]}{\bar{N}\left[i\right]}-1\right)\cdot \frac{\partial \bar{N}\left[i\right]}{\partial \theta }\right)=0. \end{array}$$

Consequently the regularity condition is fulfilled, cf. Equation (6). Proceeding with the second derivative

(12) $$\begin{array}{cc} E\left(\frac{\partial^{2}\ln p\left(\vec{x},\theta \right)}{\partial \theta^{2}}\right) & =E\left(\sum_{i=1}^{l}\frac{\partial^{2}\ln p\left(\vec{x},\theta \right)}{\partial \bar{N}{\left[i\right]}^{2}}\cdot {\left(\frac{\partial \bar{N}\left[i\right]}{\partial \theta }\right)}^{2}\right)\\ & =\sum_{i=1}^{l}\left(-\frac{1}{\bar{N}\left[i\right]}\right)\cdot {\left(\frac{\partial \bar{N}\left[i\right]}{\partial \theta }\right)}^{2} \end{array}$$

yields together with Equations (4), (5) and (7) the CRB

(13) $$\begin{array}{c} \mathrm{Var}\left(\hat{\theta }\right)\geq \frac{1}{\sum_{i=1}^{l}\frac{1}{\bar{N}\left[i\right]}\cdot {\left(\frac{\partial \bar{N}\left[i\right]}{\partial \theta }\right)}^{2}}. \end{array}$$

As a result, the CRB is the inverse of the total Fisher information, which is the sum of the Fisher information of all signal elements due to the white noise assumption. Interestingly, the Fisher information of each signal element (sample) is the inverse of the number of photons weighted with the sensitivity with respect to the unknown quantity.

For a large number of photons $\bar{N}\left[i\right]$, shot noise can be approximately described by a Gaussian distribution with the variance $\sigma_{i}^{2}=\bar{N}\left[i\right]$. In addition, the resulting signal variance is constant for a dominant (constant) background light signal. In order to describe this case and for the sake of completeness, the well-known case of additive white Gaussian noise (AWGN) with constant variance $\sigma^{2}$, i.e., for

(14) $$\begin{array}{c} p\left(\vec{x},\theta \right)=\prod_{m=1}^{l}\frac{1}{\sqrt{2\pi \sigma^{2}}}e^{-\frac{{\left(N\left[m\right]-\bar{N}\left[m\right]\right)}^{2}}{2\sigma^{2}}} \end{array}$$

is additionally described. The respective log-likelihood function is

(15) $$\begin{array}{c} \ln p\left(\vec{x},\theta \right)=\sum_{m=1}^{l}\left(-\frac{1}{2}\ln\left(2\pi \sigma^{2}\right)-\frac{{\left(N\left[m\right]-\bar{N}\left[m\right]\right)}^{2}}{2\sigma^{2}}\right). \end{array}$$

and the first derivative is

(16) $$\begin{array}{cc} E\left(\frac{\partial \ln p(\vec{x},\theta )}{\partial \theta }\right) & =E\left(\sum_{i=1}^{l}\frac{\partial \ln p(\vec{x},\theta )}{\partial \bar{N}\left[i\right]}\cdot \frac{\partial \bar{N}\left[i\right]}{\partial \theta }\right)\\ & =E\left(\sum_{i=1}^{l}\left(\frac{N\left[i\right]-\bar{N}\left[i\right]}{\sigma^{2}}\right)\cdot \frac{\partial \bar{N}\left[i\right]}{\partial \theta }\right)=0. \end{array}$$

Thus the regularity condition is fulfilled for AWGN. The second derivative of the likelihood function reads

(17) $$\begin{array}{cc} E\left(\frac{\partial^{2}\ln p\left(\vec{x},\theta \right)}{\partial \theta^{2}}\right) & =E\left(\sum_{i=1}^{l}\frac{\partial^{2}\ln p\left(\vec{x},\theta \right)}{\partial \bar{N}{\left[i\right]}^{2}}\cdot {\left(\frac{\partial \bar{N}\left[i\right]}{\partial \theta }\right)}^{2}\right)\\ & =\sum_{i=1}^{l}\left(-\frac{1}{\sigma^{2}}\right)\cdot {\left(\frac{\partial \bar{N}\left[i\right]}{\partial \theta }\right)}^{2}. \end{array}$$

After inserting the result into Equations (4), (5) and (7) the CRB is obtained in the form

(18) $$\begin{array}{c} \mathrm{Var}\left(\hat{\theta }\right)\geq \frac{1}{\sum_{i=1}^{l}\frac{1}{\sigma^{2}}\cdot {\left(\frac{\partial \bar{N}\left[i\right]}{\partial \theta }\right)}^{2}}. \end{array}$$

Again, the CRB is the inverse of the sum of the Fisher information of all signal elements due to the white noise assumption. Here the Fisher information of each signal element is the inverted noise variance weighted with the light signal sensitivity with respect to the unknown quantity.

Please note that both derivations of the CRB were conducted for a 1d signal (series), but are also applicable or can be enhanced for a 2d signal (image). Either all image elements are listed in a single vector to create a 1d signal or the image elements remain in a matrix shape $\bar{N}\in R^{lxh}$ so that a double sum occurs in Equations (13) and (18), namely

(19) $$\begin{array}{c} \mathrm{Var}\left(\hat{\theta }\right)\geq \frac{1}{\sum_{i=1}^{l}\sum_{j=1}^{h}\frac{1}{\bar{N}\left[i,j\right]}\cdot {\left(\frac{\partial \bar{N}\left[i,j\right]}{\partial \theta }\right)}^{2}}\;\mathrm{and}\;\mathrm{Var}\left(\hat{\theta }\right)\geq \frac{1}{\sum_{i=1}^{l}\sum_{j=1}^{h}\frac{1}{\sigma^{2}}\cdot {\left(\frac{\partial \bar{N}\left[i,j\right]}{\partial \theta }\right)}^{2}}. \end{array}$$

## 3. Position Measurements

Subsequently, the derived quantum shot noise limits for the laser-based position measurements of particles and surfaces are reviewed. Particles are typical measurement objects in flow measurement applications, and the surface position provides as 0d measurement a position, as 1d measurement a profile and as 2d or 3d measurement a shape of the measurement object.

### 3.1. Particle

**Figure 1:**
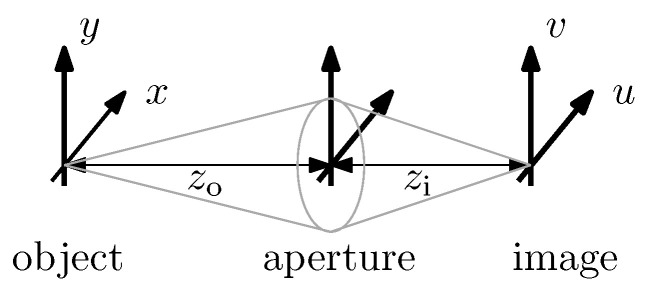
Measurement arrangement to illustrate the (*u*,*v*)-coordinates in the image plane, the (*x*,*y*)-coordinates in the object plane as well as the distances $z_{i}$, $z_{o}$ to define the absolute value of the image magnification $M=\frac{u}{x}=\frac{z_{i}}{z_{o}}$.

It is assumed that the image of a single spherical particle is recorded with a camera including possible magnification optics. Locating the particle in the recorded image is for instance essential for flow measurements based on particle tracking velocimetry (PTV). Wernet and Pline studied in 1993 the CRB of the particle position *x* or *y* (lateral to the optical axis) for a 1d Gaussian particle intensity signal with WPN [61], which was extended for a symmetric 2d Gaussian particle intensity signal by Fischer in 2013 [62]. As a result, an optimal particle image diameter ($1/e^{2}$ intensity diameter *d*) is identified as about 120% of the pixel size, which minimizes the CRB for the most disadvantageous particle position with respect to the pixel location. The CRB then reads

(20) $$\begin{array}{c} \mathrm{Var}\left(\hat{x}\right)\geq 0.22\cdot \frac{w_{\mathrm{pixel}}^{2}}{M^{2}}\cdot \frac{1}{N_{\mathrm{photon}}} \end{array}$$

with $w_{\mathrm{pixel}}$ as the pixel size, *M* as the absolute value of the image magnification and $N_{\mathrm{photon}}$ as the mean total number of photons of the particle image. The image magnification is defined as the ratio $\frac{u}{x}=\frac{z_{i}}{z_{o}}$ with the *u*-axis in the image plane and the *x*-axis in the object plane, see Figure 1. Note that the same CRB holds for the positions at the two lateral axes *x* and *y*.

Westerweel studied the same 2d signal model but for the case of AWGN with the variance $\sigma^{2}$ in 1997 and 2000 [63,64]. He identified an optimal particle image radius of about 60% of the pixel size and the respective CRB:

(21) $$\begin{array}{c} \mathrm{Var}\left(\hat{x}\right)\geq 3.7\cdot \frac{w_{\mathrm{pixel}}^{2}}{M^{2}}\cdot \frac{\sigma^{2}}{N_{\mathrm{photon}}^{2}}. \end{array}$$

**Figure 2:**
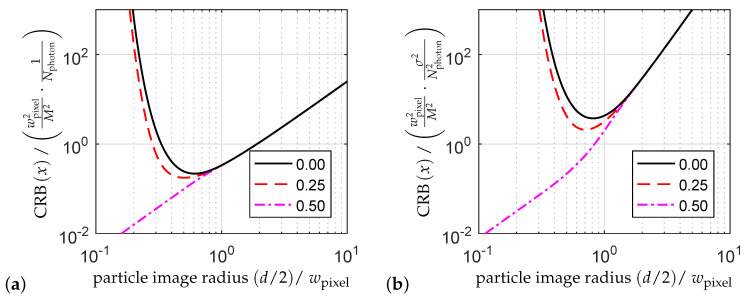
Normalized CRB for (**a**) WPN and (**b**) AWGN of the lateral particle position *x* as a function of the normalized particle image radius $\left(d/2\right)/w_{\mathrm{pixel}}$ ($1/e^{2}$ intensity radius) and for the particle positions $\left(\frac{x}{w_{\mathrm{pixel}}/M},\frac{y}{w_{\mathrm{pixel}}/M}\right)=\left(0,0\right);\left(0.25,0\right);\left(0.50,0\right)$ where (0,0) is the pixel center. The legends indicate the normalized *x*-values.

The normalized CRB as a function of the particle image radius d/2 ($1/e^{2}$ intensity radius) is shown for different particle positions with respect to the pixel and for WPN and AWGN in Figure 2a,b, respectively. For particle image sizes $d\gg w_{\mathrm{pixel}}$, the CRBs become independent of the particle position, and the CRB for WPN then reads

(22) $$\begin{array}{c} \mathrm{Var}\left(\hat{x}\right)\geq \frac{1}{16}\cdot \frac{d^{2}}{M^{2}}\cdot \frac{1}{N_{\mathrm{photon}}} \end{array}$$

and for AWGN

(23) $$\begin{array}{c} \mathrm{Var}\left(\hat{x}\right)\geq \frac{1}{10}\cdot \frac{d^{2}}{w_{\mathrm{pixel}}^{2}}\cdot \frac{d^{2}}{M^{2}}\cdot \frac{\sigma^{2}}{N_{\mathrm{photon}}^{2}}. \end{array}$$

For locating diffraction-limited spots (i.e., usually no Gaussian intensity distribution of the particle image) much larger than the pixel size, for instance for locating far distant stars, Falconi and later Lindegren derived for WPN a measurement limit in 1964 and 1978, respectively, without the framework of the CRB [65,66,67]. In 2017, Fischer presented a derivation using the CRB [68]. The general CRB result depends on the aperture of the optics, and the resulting CRB for the example of a circular aperture with the radius *r*, an object distance $z_{o}$ and the wave number k=2π/λ with the wavelength λ is

(24) $$\begin{array}{c} \mathrm{Var}\left(\hat{x}\right)\geq \frac{1}{{\left(r/z_{o}\right)}^{2}k^{2}}\cdot \frac{1}{N_{\mathrm{photon}}}. \end{array}$$

Note that the optical magnification is now limited by the diffraction limit so that a measure of the numerical aperture $N_{A}=r/z_{o}$ as well as the wavelength influence the quantum shot noise limit of the lateral position measurement. The term $\lambda /N_{A}$ is proportional to the diffraction-limited spot size, so that the CRB turns out to be directly proportional to the spot size squared. This finding is consistent with the more general CRB result shown in Figure 2a, which converges for spot sizes much larger than the pixel size. Furthermore, the dependence of the square root of the CRB from wavelength and numerical aperture is identified to be identical with the dependence of the Abbe’s resolution limit [69].

**Figure 3:**
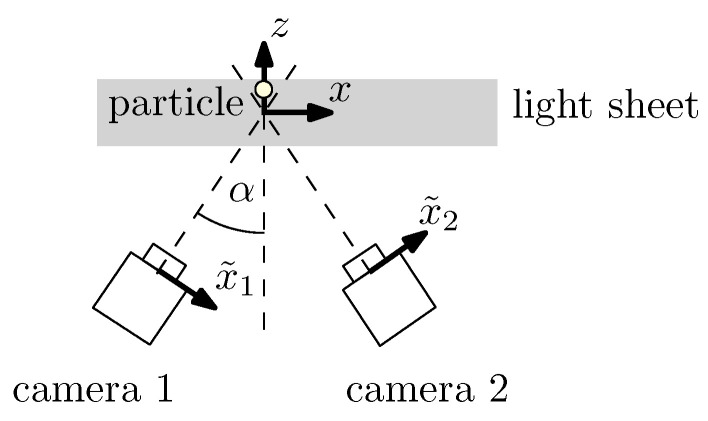
Symmetric measurement arrangement to measure the 3d particle position with a stereoscopic approach (triangulation). Please note that the *y*-position, which is perpendicular to the *z*- and the *x*-axis, is neglected here.

In order to calculate the CRB for the axial *z*-position of a particle, a symmetric stereoscopic approach, i.e., a triangulation with two tilted cameras is considered as shown in Figure 3. Assuming a light sheet thickness much smaller than the viewing distance, the *z*-position is calculated from the two measured image positions ${\tilde{x}}_{1}$ and ${\tilde{x}}_{2}$ (neglecting the $\tilde{y}$-position for the sake of simplicity), the camera tilting angle α and the image magnification *M* using basic geometry:

(25) $$\begin{array}{c} z=\frac{1}{2M\sin\alpha }\left({\tilde{x}}_{1}-{\tilde{x}}_{2}\right). \end{array}$$

By applying an uncertainty propagation calculation, by taking the noise independence of the two camera signals into account and by inserting the CRB result from Equation (22) for the two particle positions in the cameras’ coordinate systems ${\tilde{x}}_{1}=Mx_{1}$, ${\tilde{x}}_{2}=Mx_{2}$, the CRB of the axial particle position is obtained in the form

(26) $$\begin{array}{c} \mathrm{Var}\left(\hat{z}\right)\geq \frac{1}{16}\cdot \frac{d^{2}}{M^{2}\sin^{2}\alpha }\cdot \frac{1}{N_{\mathrm{photon}}}. \end{array}$$

For comparison reasons, $N_{\mathrm{photon}}$ is here the sum of all detected photons from both camera images. As a result, the CRB for the axial particle position is, except for the increase by the sine of the tilt angle α, identical with the CRB for the lateral particle position, see Equation (22). Please note that the tilt angle is always between $0^{\circ }$ and $90^{\circ }$, and the absolute value of the sine is always smaller than one. Note also that the CRB notation in Equation (26) includes an expectable dependence from the axial distance between particle and cameras, which is hidden in the camera tilting angle α (considering a constant camera distance and a varying light sheet position) and the optical magnification *M*. While a symmetric measurement configuration was considered here, the calculation can also be adapted for other stereoscopic or even multi-camera approaches as well as for the remaining particle coordinates [70,71].

In order to measure the axial position of a particle, time-of-flight is also a well known technique where half of the light traveling time *t* to and from the scattering particle is evaluated and multiplied by the light velocity *c*:

(27) $$\begin{array}{c} z=\frac{t}{2}\cdot c. \end{array}$$

This measurement principle is used, for instance, in Light Detection And Ranging (LiDAR) systems. Assuming a Gaussian intensity light pulse with the $1/e^{2}$-width $d_{z}$, adapting the result from Equation (22) for the time axis instead of the spatial axis and then multiplying the result with half of the light speed squared leads to the following CRB of the axial position for time-of-flight measurements:

(28) $$\begin{array}{c} \mathrm{Var}\left(\hat{z}\right)\geq \frac{{\left(d_{z}/2\right)}^{2}}{16}\cdot \frac{1}{N_{\mathrm{photon}}}. \end{array}$$

As a result, the CRBs for time-of-flight and triangulation seem to have a similar form, see Equation (26). Due to the axial measurement, however, the lateral image magnification and the tilt angle are missing here, because both have no meaning in the time-of-flight measurement concept. As a result, the aforementioned dependence of the CRB from particle distance for triangulation does not occur for time-of-flight measurements. Using the frequency-time uncertainty for a Gaussian pulse over time, i.e., $\sigma_{t}\cdot \sigma_{f}=\frac{1}{2\pi }$, the time-space relation to yield $\sigma_{t}c=d_{z}/4$, and propagating the frequency uncertainty to the wave number uncertainty $\sigma_{k}=\sigma_{f}\frac{2\pi }{c}$, it follows $d_{z}=4/\sigma_{k}$ and thus

(29) $$\begin{array}{c} \mathrm{Var}\left(\hat{z}\right)\geq \frac{1}{4\mathrm{Var}\left(k\right)}\cdot \frac{1}{N_{\mathrm{photon}}} \end{array}$$

with $\mathrm{Var}\left(k\right)=\sigma_{k}^{2}$ as the wave number variance due to the spectral width (or wavelength distribution) of the Gaussian laser pulse.

### 3.2. Surface

Measuring with lasers on rough surfaces leads to speckles, which enables lateral position measurements without modifying the surface [72]. Note that speckles allow a surface position measurement only relative to an initial position of the same surface element. Otherwise the speckle pattern correlation decreases and the measurement uncertainty increases due to speckle noise. For ideal speckle pattern correlation, the CRB of the lateral position measurement for a large number (>10) of fully developed speckles and WPN was derived by Fischer in 2017 by neglecting the pixel discretization [68]. The results depend on the aperture of the optical system. The derived analytic CRB expression for a circular aperture with radius *r* and an object distance $z_{o}$ from the lens reads

(30) $$\begin{array}{c} \mathrm{Var}\left(\hat{x}\right)\geq \frac{2}{{\left(r/z_{o}\right)}^{2}k^{2}}\cdot \frac{1}{N_{\mathrm{photon}}}=\frac{1}{2\pi }\cdot \frac{s_{\mathrm{speckle}}^{2}}{M^{2}}\cdot \frac{1}{N_{\mathrm{photon}}}, \end{array}$$

with the speckle size $s_{\mathrm{speckle}}=\frac{\lambda \cdot z_{i}}{\sqrt{\pi r^{2}}}$ [72] in the image plane, the axial distance $z_{i}=M\cdot z_{o}$ from the image plane to the lens plane (see Figure 1) and the wave number k=2π/λ.

As a result, the CRB for speckles is, aside from a factor of 2, identical to the CRB for a single light spot, cf. Equation (30) with Equation (24). Here, the speckle size is identified as the characteristic scale, which again is a result of the diffraction limit. Recently in 2018, Tausendfreund et al. evaluated the more general CRB result numerically to take pixel discretization into account [73]. The CRB then increases for a speckle size smaller than the pixel size as expected.

In order to determine the CRB for WPN also for partially developed speckles or non-speckled images, a numerical evaluation of Equation (19) needs to be performed as is demonstrated in [68]. In addition, a recent tutorial from Chao et al. [74] is recommended for this purpose, where the consideration of other measurands is also included.

The laser-based axial position measurement of rough surfaces can be performed with laser triangulation, where triangulation here refers to a classical laser triangulation sensor with a surface normal illumination and a single camera. In fact, triangulation only works on rough surfaces. However, triangulation is not limited by quantum shot noise but by speckle noise, which was derived by Dorsch et al. in 1994 [75]. The uncertainty was found to be essentially the same as if the measurement would be done with one single photon and obeys Equation (24) with $N_{\mathrm{photon}}=1$. A light source with reduced coherence is required to overcome this serious limitation. Concerning white-light interferometry on rough surfaces, a CRB-based study of the achievable measurement uncertainty was performed by Pavliček and Hýbl in 2012 but for a Gaussian noise model [76]. However, according to Häusler 2005 [77], the measurement uncertainty is typically independent from the number of photons and does not scale with the aperture, but is (up to a factor around unity) the surface roughness.

If the surface is optically smooth or a mirror is attached to the measurement object, i.e., no speckles occur, interferometry and confocal microscopy are common measurement techniques for the axial position. The early work of Ingelstam from 1960 is mentioned at first, because he was possibly among the first to find an uncertainty relation for interferometry [78]. In 2014, Pavliček and Häusler derived the CRBs for interferometry and confocal microscopy considering WPN [79]. The CRB for classical interferometry such as with a Michelson interferometer reads

(31) $$\begin{array}{c} \mathrm{Var}\left(\hat{z}\right)\geq \frac{1}{4k^{2}}\cdot \frac{1}{N_{\mathrm{photon}}}, \end{array}$$

which is according to [79] lower than the CRB of white-light interferometry. For confocal microscopy the CRB is

(32) $$\begin{array}{c} \mathrm{Var}\left(\hat{z}\right)\geq \frac{10}{{\mathrm{NA}}^{2}k^{2}}\cdot \frac{1}{N_{\mathrm{photon}}}, \end{array}$$

with NA as numerical aperture. Finally, Pavliček et al. found a more general form of the fundamental uncertainty principle for axial position measurements on smooth surfaces, which reads [79,80]

(33) $$\begin{array}{c} \mathrm{Var}\left(\hat{z}\right)\geq \frac{1}{4\mathrm{Var}\left(k_{z}\right)}\cdot \frac{1}{N_{\mathrm{photon}}}. \end{array}$$

Hence, the uncertainty of the position *z* becomes minimal if the variance of the respective wave vector component $k_{z}$ in the optical setup is maximal. Note the variance of the wave number occurs due to the different light ray directions, while the laser wavelength is fix. This explains the lower CRB for classical interferometry in comparison with the CRB for confocal microscopy, because the variance of the wave vector components is maximized in a classical interferometry setup (two rays with opposite directions).

Regarding time-of-flight measurements, the result from Equation (29) is also applicable for measurements on smooth surfaces. Hence, the CRB result for interferometry in Equation (33) is almost identical with Equation (29) for time-of-flight measurements. However, the fundamental difference is that time-of-flight measurements make use of the wavelength uncertainty while interferometry and confocal microscopy make use of the light ray direction uncertainty, although both belongs to the wave vector uncertainty and thus to the photon momentum uncertainty.

A similar uncertainty principle of Equation (33) also holds regarding a lateral position measurement along the *x*-axis and the wave vector component $k_{x}$. This case occurs for instance for deflectometry (on smooth surfaces) when locating the light spot on the camera plane. The resulting measurement uncertainty limit of the surface angle α for deflectometry was derived by Pavliček and Häusler in 2014 [79] and is mentioned here for the sake of completeness:

(34) $$\begin{array}{c} \mathrm{Var}\left(\hat{\alpha }\right)\geq \frac{1}{D^{2}k^{2}}\cdot \frac{1}{N_{\mathrm{photon}}}, \end{array}$$

where *D* denotes the waist diameter ($1/e^{2}$ width) of the beam located on the surface. When *D* is considered as a lateral resolution distance on the surface, Equation (34) represents an uncertainty principle between the angle and the lateral position. More precisely, it links the angular uncertainty with the lateral resolution. Since the product $\frac{D}{2}\cdot \sqrt{\mathrm{Var}\left(\alpha \right)}$ is the uncertainty in the axial direction within the turning points of the beam spot profile on the surface, the CRB for the axial position reads

(35) $$\begin{array}{c} \mathrm{Var}\left(\hat{z}\right)\geq \frac{1}{4k^{2}}\cdot \frac{1}{N_{\mathrm{photon}}}, \end{array}$$

which is equal to the result for the classical interferometry, see Equation (31). Note, however, that deflectometry allows surface angle measurements and the determination of a height profile requires reconstruction algorithms.

## 4. Displacement, Strain and Velocity Measurements

The definition of a displacement in *x*-direction is

(36) $$\begin{array}{c} \Delta x=x_{2}-x_{1}. \end{array}$$

Since WPN is considered, the two position measurements $x_{1}$, $x_{2}$ are uncorrelated and thus

(37) $$\begin{array}{c} \mathrm{CRB}\left(\Delta x\right)=\mathrm{CRB}\left(x_{2}\right)+\mathrm{CRB}\left(x_{1}\right). \end{array}$$

As a result, all findings concerning the CRB of the position measurement from Section 3 can be directly applied to yield the CRB for displacement measurements. Assuming identical measurement conditions for the two position measurements and using Equation (24), the CRB for the lateral displacement measurements is obtained as

(38) $$\begin{array}{c} \mathrm{Var}\left(\Delta \hat{x}\right)\geq \frac{2}{{\left(r/z_{o}\right)}^{2}k^{2}}\cdot \frac{1}{N_{\mathrm{photon}}}. \end{array}$$

Regarding the analogous derivation of the CRB for the axial displacement measurement, it follows with Equation (29)

(39) $$\begin{array}{c} \mathrm{Var}\left(\Delta \hat{z}\right)\geq \frac{1}{2\mathrm{Var}\left(k\right)}\cdot \frac{1}{N_{\mathrm{photon}}}. \end{array}$$

Using the displacement Δx of a surface element, the *x*-component of a local strain in a strain field is with respect to a reference length $L_{x}$ [81]

(40) $$\begin{array}{c} s_{x}=\frac{\Delta x}{L_{x}}. \end{array}$$

Note that only one strain component is considered here as an example, and a more rigorous derivation of the three dimensional strain field from the displacement field or the spatial gradients of the displacement field, respectively, can be studied in [82,83]. According to the definition in Equation (40), the CRB of the strain component $s_{x}$ reads

(41) $$\begin{array}{c} \mathrm{CRB}\left(s_{x}\right)=\frac{1}{L_{x}^{2}}\cdot \mathrm{CRB}\left(\Delta x\right), \end{array}$$

which can be solved with Equation (38). Note that an analogous CRB relation is obtained for the *z*-component of the strain, where Equation (39) has to be applied.

For a non-accelerated movement, the velocity component in *x*-direction is

(42) $$\begin{array}{c} v_{x}=\frac{\Delta x}{T}, \end{array}$$

with the temporal distance *T* between the two position measurements. Note that the velocity is defined as the derivative of the displacement with respect to time, while the strain is defined as the derivative of the displacement with respect to space. Note further that only a single velocity component is considered here, because the findings hold for each of the three velocity components. For the velocity component defined in Equation (42), the CRB follows as

(43) $$\begin{array}{c} \mathrm{CRB}\left(v_{x}\right)=\frac{1}{T^{2}}\cdot \mathrm{CRB}\left(\Delta x\right) \end{array}$$

and the solution is obtained by inserting Equation (38). Again, an analogous derivation and the usage of Equation (39) leads to the CRB for the velocity component in *z*-direction.

Common laser-based velocity measurement principles can be divided into principles based on the time-of-flight and those based on the Doppler effect, or the position and the frequency property of the photon, respectively.

Concerning a topical review of the different flow velocity measurement techniques based on the measurement of particle velocities, see [10]. Considering particle velocity measurements, the previous derivation of the CRB covers time-of-flight velocity measurement techniques based on particle position measurements such as particle tracking velocimetry (PTV) [84,85] and particle image velocimetry (PIV) [86,87]. The CRB for PTV with respect to WPN was considered by Wernet in 1993 [61] and Fischer in 2013 [62]. Instead of measuring the particle displacement Δx for a given temporal distance, time-of-flight velocity measurement techniques based on measuring the temporal distance *T* for a given particle displacement also exist, namely laser-two-focus velocimetry (L2F) [88,89] and the related imaging technique spatial filter velocimetry (SFV) [90,91]. For L2F, Oliver et al. performed an error propagation calculation for Poisson statistics in 1980 [92], and Lading and Jørgensen derived the CRB in 1983 and 1990 [93,94]. Further CRB calculations for L2F are contained in [95,96].

The most common Doppler velocity measurement technique for particles in flows is laser Doppler anemometry (LDA) [97,98], for which CRB studies were performed by several groups regarding AWGN [99,100,101,102]. The CRB for WPN is derived by Fischer et al. in 2010 and 2016 [95,96]. Instead of using light mixing to measure the Doppler frequency as in LDA, intensity-based absolute light frequency measurements using optical filters also exist and are known as Doppler global velocimetry (DGV) and planar Doppler velocimetry (PDV) [103,104,105,106,107]. McKenzie studied in 1996 the shot noise limit of DGV using an error propagation calculation [108]. Fischer et al. investigated the CRB for WPN covering DGV techniques with and without laser frequency modulation in 2008 and 2010 [95,109,110]. Finally, an overview and summary of all DGV techniques including the CRB for WPN is given by Fischer in 2017 [111].

Using the CRB approach, a comparison of the quantum shot noise limit of L2F, LDA and DGV was performed by Fischer and Czarske in 2010 [95], and, by including PTV/PIV, completed by Fischer in 2013 [62]. Finally, Fischer proved in 2016 that time-of-flight and Doppler techniques (except for DGV due to the light attenuating filter) attain identical CRBs of the form [96]:

(44) $$\begin{array}{c} \mathrm{CRB}\left(v\right)∼\frac{b^{2}}{L^{2}}\frac{T}{L}{\left|v\right|}^{3}\frac{1}{N_{\mathrm{photon}}}. \end{array}$$

Please note that the result applies for a single particle with the velocity component *v* crossing the measurement volume with the characteristic dimensional extension (or spatial resolution) *L* in the (velocity dependent) measurement time (or temporal resolution) *T*, where the illuminating light is distributed over space and is concentrated in peaks with the characteristic width *b*. Averaging over a given measurement time $T_{m}\gg T$, multiple particles occur and the number of particles is directly proportional to *v*. Then the CRB becomes directly proportional to the velocity squared, i.e., $\mathrm{CRB}\left(v\right)∼{\left|v\right|}^{2}$.

Similar CRB studies for surface velocity measurements are not as far as for particle velocity measurement techniques, mainly due to the limiting speckle effect. When the LDA principle is applied to obtain Doppler surface velocity measurements, the technique is termed laser Doppler velocimetry (LDV), and the speckle effect was identified to influence the resulting CRB [112]. Concerning lateral time-of-flight surface velocity measurements based on speckle displacement measurements, Equation (43) can be applied.

## 5. Conclusions and Outlook

The Cramér-Rao bound (CRB) is presented as a useful tool in order to determine the quantum shot noise limits of laser-based measurement systems. The CRB is the inverse of the Fisher information and the Cramér-Rao inequality represents an entropic uncertainty relation. Since the CRB yields the minimal achievable measurement uncertainty squared, the CRB is derived for light signals superposed by white Poissonian noise, and the specific CRB results for the different particle and surface position measurement techniques are reviewed. As a result, many measurement techniques exhibit a similar or even the same CRB, which is indirectly proportional to the total number of detected photons and indirectly proportional to the wave number squared.

The CRB results for white Poissonian noise are also reviewed regarding displacement, strain and velocity measurements of particles and surfaces, respectively. In particular the particle-based flow velocity measurements, time-of-flight and Doppler measurement principles without light attenuation or absorption are limited by similar lower bounds. The CRBs are indirectly proportional to the total number of photons and directly proportional to the flow velocity when multiple particles cross the measurement volume in a given measurement time.

As a consequence, the CRB investigations reveal that most of the measurement techniques perform similarly with respect to white Poissonian noise, also for complementary principles such as time-of-flight and Doppler particle velocity measurements as well as time-of-flight and interferometry position measurements. Since white Poissonian noise is an optimal quantum state minimizing Heisenberg’s uncertainty principle, the derived measurement limits using the CRB are in agreement with the measurement limits that follow from Heisenberg’s uncertainty principle. This was recently demonstrated for position measurements [68,79] and flow velocity measurements [96]. As a result, the CRB approach enables, with a formal calculation procedure, to derive and compare the fundamental quantum-mechanical measurement limits of laser-based measurement techniques.

It is important to note that quantum shot noise is only one contribution to the measurement uncertainty budget, and the performance of the different measurement techniques differ mostly with respect to the additional uncertainty contributions. Furthermore, the cross-sensitivities with respect to influences from the measurement environment as well as the realizability of measurement system setups with available technology are key factors in selecting the suitable measurement approach. Random and systematic errors can be considered within the framework of the CRB in agreement with the international guide to the expression of uncertainty in measurement (GUM) [33]. Therefore, future comparative studies of the limits of different measurement techniques should incorporate further sources of uncertainty in addition to photon shot noise. Since the CRB states a lower bound but not the existence of an efficient estimation algorithm that attains the CRB, future studies should also consider the estimation efficiency of different signal processing strategies. The CRB investigation (demonstrated here for the laser-based measurement of position, displacement, strain and velocity) should also be conducted for further measurands, in order to deepen the understanding of the potentials of existing laser-based measurements, to design novel optimized measurement systems and, thus, to achieve a progress in laser metrology.
